# Supplementary material for: Programmed genome editing of the omega-1 ribonuclease of the blood fluke, Schistosoma mansoni
Source: eLife. 2019 Jan 15;8:e41337. doi: 10.7554/eLife.41337 (PMC6355194; doi:10.7554/eLife.41337)
Supplement: Transparent reporting form [file elife-41337-transrepform.docx]

***eLife’s* transparent reporting form**

We encourage authors to provide detailed information *within their submission* to facilitate the interpretation and replication of experiments. Authors can upload supporting documentation to indicate the use of appropriate reporting guidelines for health-related research (see [EQUATOR Network](http://www.equator-network.org/%20)), life science research (see the [BioSharing Information Resource](https://biosharing.org/)), or the [ARRIVE guidelines](http://www.plosbiology.org/article/info:doi/10.1371/journal.pbio.1000412) for reporting work involving animal research. Where applicable, authors should refer to any relevant reporting standards documents in this form.

If you have any questions, please consult our Journal Policies and/or contact us: [editorial@elifesciences.org](mailto:editorial@elifesciences.org).

**Sample-size estimation**

- You should state whether an appropriate sample size was computed when the study was being designed
- You should state the statistical method of sample size computation and any required assumptions
- If no explicit power analysis was used, you should describe how you decided what sample (replicate) size (number) to use

Please outline where this information can be found within the submission (e.g., sections or figure legends), or explain why this information doesn’t apply to your submission:

Sample size estimation were not undertaken in the report because such an approach was not relevant to the questions that we posed in this study and the assays undertaken to answer the questions. One the possible exception was the analysis of circumoval granuloma size of wild type vs omega-1 knockout schistosome eggs, the findings for which are presented in Figure 5. Here however, two biological repeats were undertaken, with more than 100 granuloma were measured and counted in each of the control and experimental groups. The ranges of the values for the control and experimental groups did not overlap – these ranges were so divergent that Welch’s unequal variance t-test (rather than Student’s t-test) was used in the statistical analysis. This finding (in hindsight) obviates concern about the absence of power calculations on sample size to undertaken during design of the experiment.

**Replicates**

- You should report how often each experiment was performed
- You should include a definition of biological versus technical replication
- The data obtained should be provided and sufficient information should be provided to indicate the number of independent biological and/or technical replicates
- If you encountered any outliers, you should describe how these were handled
- Criteria for exclusion/inclusion of data should be clearly stated
- High-throughput sequence data should be uploaded before submission, with a private link for reviewers provided (these are available from both GEO and ArrayExpress)

Please outline where this information can be found within the submission (e.g., sections or figure legends), or explain why this information doesn’t apply to your submission:

Two or more (up to eleven) biological replicates of each experiment were performed. We included a definition of biological versus technical replicates in the Materials & Methods section, as follows:

‘In the assays above, we performed two or more biological replicates. These biological replicates represented parallel measurements of biologically discrete samples in order to capture any random biological variation. Technical replicates were undertaken as well; these represented two or three repeated measurements of the same sample undertaken as independent measurements of the random noise associated with the investigator, equipment or protocol’.

High throughput sequence data are available, as indicated in the Materials and Methods section, at both ENA and NCBI:

# ‘GenBank/EMBL/DDBJ

***Database accessions*** Sequence reads from the amplicon NGS libraries are available at the European Nucleotide Archive, study accession number ERP110149. Additional information is available at Bioproject PRJNA415471, <https://www.ncbi.nlm.nih.gov/bioproject/PRJNA415471> and GenBank accessions SRR6374209, SRR6374210’.

‘.

**Statistical reporting**

- Statistical analysis methods should be described and justified
- Raw data should be presented in figures whenever informative to do so (typically when N per group is less than 10)
- For each experiment, you should identify the statistical tests used, exact values of N, definitions of center, methods of multiple test correction, and dispersion and precision measures (e.g., mean, median, SD, SEM, confidence intervals; and, for the major substantive results, a measure of effect size (e.g., Pearson's r, Cohen's d)
- Report exact p-values wherever possible alongside the summary statistics and 95% confidence intervals. These should be reported for all key questions and not only when the p-value is less than 0.05.

Please outline where this information can be found within the submission (e.g., sections or figure legends), or explain why this information doesn’t apply to your submission:

These details have been provided in the Results, including in figure legends, for example, in the legends of Figures 4 and 5|:

‘**Figure 4.** Reduced Th2 cytokine levels following exposure to Δω1-SEA. Reduction in Th2 cytokines IL-4 and IL-5 but not IL-13 following pulsing of Mϕ (PMA induced-THP-1 cells) with Δω1-SEA prior to co-culture with human CD4^+^ T cells (Jurkat cell line) compared with WT-SEA pulsed-Mϕ (top panels). In addition, levels of IL-6 and TNF-⍺ were reduced where Mϕ were first pulsed with Δω1-SEA but not WT SEA. Differences were not evident for IL-10. The assay was carried out in triplicate; *p* < 0.0001, ≤ 0.0001, 0.0038 and 0.0252 indicated as ****, ***, ** and *, respectively (one-way ANOVA, multiple comparison, *n* = 4).

**Figure 5.** Pulmonary circumoval granulomas revealed attenuated granulomatous response to Δω1 schistosome eggs. Schistosome eggs (~3,000 eggs) that had been transduced with lentivirus virions encoding ω1*-*specific sgRNA and Cas9 in tandem with ssODN were introduced via the tail vein into mice. The mice were euthanized 10 days later; thin sections of the left lung were stained with H&E, and circumoval granulomas counted and measured. **A,** Representative 2D scanned micrographs of granulomas inoculated with WT eggs (2× magnification) and 20× magnification (A1 and A2), and with Δω1 eggs; **B**, (2×), B1 and B2 (20×). C, control mouse lung. **D** and **E**, Representative micrograph of individual, control eggs induced-granuloma that was counted to assess for granuloma volume. **F** and **G**, Representative micrographs showing Δω1 egg induced-granulomas. All single egg induced-granuloma from WT and Δω1 eggs were measured and granuloma volume estimated. **H**, Scatter plots of the volume (mm^3^) for individual granuloma, mean ± SE (red) are shown. The volumes of granulomas induced by Δω1 eggs were significantly smaller than those surrounding WT eggs (Welch’s *t*-test, *p* ≤ 0.0001, *n* >100)’.

(For large datasets, or papers with a very large number of statistical tests, you may upload a single table file with tests, Ns, etc., with reference to sections in the manuscript.)

**Group allocation**

- Indicate how samples were allocated into experimental groups (in the case of clinical studies, please specify allocation to treatment method); if randomization was used, please also state if restricted randomization was applied
- Indicate if masking was used during group allocation, data collection and/or data analysis

Please outline where this information can be found within the submission (e.g., sections or figure legends), or explain why this information doesn’t apply to your submission:

Allocation of samples

Material and Methods section:

**‘Schistosome egg-induced primary pulmonary granulomas**

For induction of circumoval, egg-induced granulomas in the lungs of mice, 8 week old female (28) BALB/c mice were injected with 3,000 WT eggs or Δω1-eggs (from experiment pLV-ω1X6 with ssODN) or 1×PBS as negative control by tail vein, as described (62). The mice were euthanized 10 days later. Each group included 3 or 5 mice, two biological replicates were undertaken, totaling 6-10 mice for each treatment group. Before starting the experiment, mice were allocated randomly to the control or experimental treatment groups’.

**Additional data files (“source data”)**

- We encourage you to upload relevant additional data files, such as numerical data that are represented as a graph in a figure, or as a summary table
- Where provided, these should be in the most useful format, and they can be uploaded as “Source data” files linked to a main figure or table
- Include model definition files including the full list of parameters used
- Include code used for data analysis (e.g., R, MatLab)
- Avoid stating that data files are “available upon request”

Please indicate the figures or tables for which source data files have been provided:

Figure 3 panels B and D; and Figure 3— figure supplement 1 provide all the source data on the figures themselves. Each data point, which represents the value obtained in a separate biological replicate, is provided in the image.
